# *In vitro* and intrathecal siRNA mediated K_V_1.1 knock-down in primary sensory neurons

**DOI:** 10.1016/j.mcn.2011.08.007

**Published:** 2011-11

**Authors:** Mark D. Baker, Ya-Chun Chen, Syed U. Shah, Kenji Okuse

**Affiliations:** a Queen Mary University of London, Barts and The London School of Medicine and Dentistry, Neuroscience and Trauma Centre, Blizard Institute of Cell and Molecular Science, 4 Newark Street, London E1 2AT, UK; b Division of Cell and Molecular Biology, Faculty of Natural Sciences, Imperial College London, London SW7 2AZ, UK

**Keywords:** Sensory neuron, Axon, Potassium channel, Dendrotoxin-K, RNA interference, QPCR

## Abstract

K_V_1.1 is a *Shaker* homologue K^+^ channel that contributes to the juxta-paranodal membrane conductance in myelinated axons, and is blocked by fampridine (4-aminopyridine), used to treat the symptoms of multiple sclerosis. The present experiments investigate K_V_1.1 function in primary sensory neurons and A-fibres, and help define its characteristics as a drug-target using sequence specific small-interfering RNAs (siRNAs). siRNA (71 nM) was used to knock-down functional expression of K_V_1.1 in sensory neurons (> 25 μm in apparent diameter) in culture, and was also delivered intrathecally *in vivo* (9.3 μg). K^+^ channel knock-down in sensory neurons was found to make the voltage-threshold for action potential generation significantly more negative than in control (*p* = 0.02), led to the breakdown of accommodation and promoted spontaneous action potential firing. Exposure to dendrotoxin-K (DTX-K, 10–100 nM) also selectively abolished K^+^ currents at negative potentials and made voltage-threshold more negative, consistent with K_V_1.1 controlling excitability close to the nominal resting potential of the neuron cell body, near − 60 mV. Introduction of one working siRNA sequence into the intrathecal space *in vivo* was associated with a small increase in the amplitude of the depolarising after-potential in sacral spinal roots (*p* < 0.02), suggesting a reduction in the number of working K^+^ channels in internodal axon membrane. Our study provides evidence that K_V_1.1 contributes to the control of peripheral sensory nerve excitability, and suggests that its characteristics as a putative drug target can be assessed by siRNA transfection in primary sensory neurons *in vitro* and *in vivo*.

## Introduction

Agents that block voltage-gated K^+^ (K_V_) channels in axons have proved very useful in elucidating axonal physiology, for example providing functional evidence regarding the non-homogeneous distribution of different channel sub-types in myelinated nerve (e.g. Bostock et al., 1981; Baker et al., 1987; Gordon et al., 1988). Block of axonal K^+^ channels normally covered by myelin may also be clinically useful and attempts have been made to assess this. 4-aminopyridine (4-AP; fampridine) is a small-molecule blocker of K^+^ channels, and evidence suggests that it has utility in treating symptoms of multiple sclerosis, including fatigue and visual signs (e.g. Stefoski et al., 1991; Bever et al., 1994; Rossini et al., 2001), although with a restricted range of useful concentrations because of dose-related side effects. The possible usefulness of 4-AP in diseased nerve was predicted following studies on the effect of the blocker on action potential duration following experimental demyelination (Sherratt et al., 1980; Bostock et al., 1981), where block of kinetically fast axonal K^+^ channels was found to prolong action potentials at widened nodes, thus increasing the safety factor for conduction. Dendrotoxins from black and green mamba (Genus: *Dendroaspis*) have also been used in the pre-clinical pharmacological discrimination of K_V_1 channel subtypes, where, for example, the presence of K_V_1.1 within a channel multimer allows the block of the channel by dendrotoxin-K (DTX-K, from *Dendroaspis polylepis polylepis*) at single nanomolar concentrations (Robertson et al., 1996; Wang et al., 1999).

K_V_1.1 is encoded by the gene *Kcna1*, and is a *Shaker* homologue, contributing to delayed rectifier K^+^ currents in axons, cell bodies and dendrites (Wang et al., 1994). The exquisite targeting of K_V_1.1, and K_V_1.2 to the juxta-paranode in mature myelinated nerve fibres has been demonstrated immunohistochemically (Wang et al., 1993; Rasband et al., 1999; reviewed by Rasband and Trimmer, 2001). Although K_V_1.1 is thought to function within heteromultimeric channels, coalesced with related and functionally similar sub-units (provided by K_V_1.2 in the juxtaparanodes, Wang et al., 1993) global null-mutation has also confirmed the importance of the channel in the functioning of the nervous system. Deletion of the channel underlies a form of rodent temporal lobe epilepsy (Smart et al., 1998; Wenzel et al., 2007), and the loss of control of axonal excitability close to the motor end-plate, resulting in cold-induced neuromyotonia in young mice (Zhou et al., 1998). This latter finding highlights the non-redundant character of K_V_1.1 in the control of motor nerve excitability and is consistent with the older data on the 4-AP modifiability of internodal membrane resistance (e.g. Baker et al., 1987). Brew et al. (2003) reported that K_V_1.1 functions to generate a low-threshold K^+^ current in auditory neurons of the medial nucleus of the trapezoid body (MNTB), where it makes an important contribution to the input conductance over the membrane potential range between − 50 and − 60 mV. Channel knock-out reduced current-threshold in MNTB neurons and enhanced repetitive firing. However, the functional role of K_V_1.1 in sensory neuron cell bodies has been less well defined.

More recently, RNA interference has been applied to primary sensory neurons using lipofection (e.g. Dong et al., 2007; Shao et al., 2009) and this technology has brought with it the possibility that selective functional knock-down of a channel could be achieved by transient exposure to siRNA, rather than the use of a non-specific small molecule blocker, allowing the study of channel sub-type function by mRNA degradation and contributing to more accurate drug target characterisation. There is evidence that siRNA can be effective *in vivo*, with receptor knock-down in the dorsal root ganglia or spinal cord (e.g. Luo et al., 2005; Dong et al., 2007) and such studies may also inform future work involving viral transformation of neurons. The aim of the present work was therefore to test the hypothesis that siRNA targeting the K_V_1.1 channel in primary sensory neurons could alter neuronal excitability *in vitro*, and then subsequently to look for changes in sensory axon behaviour that may be consistent with siRNA activity *in vivo* within sacral dorsal root ganglia by recording the amplitude of the depolarising after potential (DAP) in spinal sensory roots. An advantage of selecting K_V_1.1 as a target (at least *in vitro*) is that the functional effects of siRNA can be compared and contrasted with the action of the selective polypeptide blocker, DTX-K, following acute application. *In vivo*, however, the effects of RNAi may be compared with the action of 4-AP, to avoid the presumed low myelin penetrability of the toxin.

## Results

### Effects of DTX-K

**Fig. 1:**
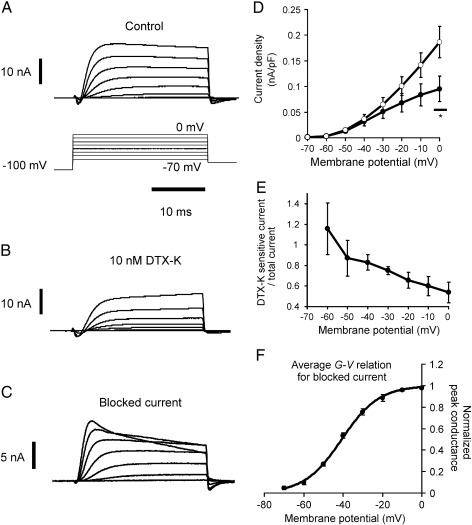
DTX-K blocks a kinetically fast, delayed rectifier K^+^ current operating at − 60 mV, in neurons > 25 μm in apparent diameter. Panels (A), (B) and (C) are families of whole-cell K^+^ currents evoked by the incrementing clamp-steps shown in (A), lower panel. (A) Control currents, (B) after exposure to 10 nM DTX-K, and (C) current blocked by DTX-K, i.e. (A)–(B). (D) Peak current density *versus* membrane potential relation for K^+^ currents in 6 neurons (○), and relation derived for those currents blocked by DTX-K in the same neurons (●, 100 nM, *n* = 2; 10 nM *n* = 4; at 0 mV, *p* = 0.021 *, paired *t*-test). (E) DTX-K sensitive component as a fraction of total K^+^ current, plotted against membrane potential, reveals a trend to lower DTX-K sensitivity with depolarisation ( *p* = 0.076, one-way, repeated-measures ANOVA). (F) Average normalised conductance *versus* membrane potential (*G*–*V*) relation for the DTX-K sensitive component (*n* = 6), taking *E*_K_ as − 102.3 mV. Smooth curve is a Boltzmann relation drawn according to best-fit parameters (*V*_1/2_ = 40.67 mV, slope factor *k* = 9.43 mV). Data plotted as mean ± standard error, errors sometimes smaller than symbol size.

DTX-K at 10 nM was found to block a low-threshold fast delayed rectifier type K^+^ current similar to IK_L_ (Brew et al., 2003) in auditory neurons (Fig. 1A–D). Estimates of the fractional block of the total K^+^ current, for example at − 60 and 0 mV, are 1.16 ± 0.17 and 0.54 ± 0.10 (Fig. 1E), with the data consistently indicating less block with increasing depolarisation, caused by recruitment of other K^+^ channel sub-types. Essentially, 87% or more of the K^+^ current at − 50 and − 60 mV is DTX-K sensitive and therefore probably involves K_V_1.1. The loss of this current, activating close to − 60 mV (Fig. 1F), helps explain why the voltage-threshold for action potential induction can move more negative when the mRNA for K_V_1.1 is down-regulated (see below).

### Sequence specific K_V_1.1 siRNA changes neuronal excitability

**Fig. 2:**
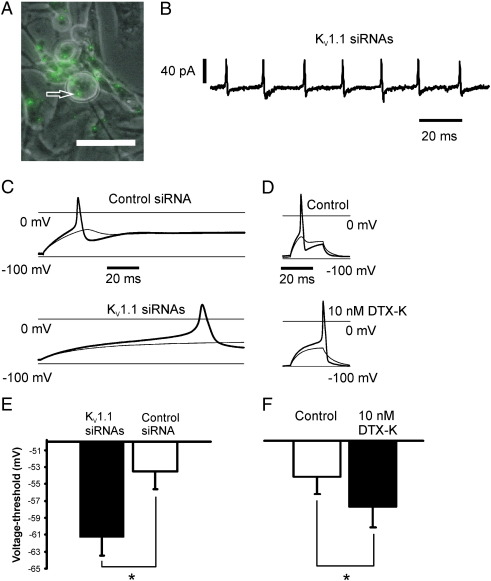
Transfection of 3 sequence specific siRNAs targeting K_V_1.1, and exposure to 10 nM DTX-K cause similar changes in the excitability of primary sensory neurons in culture. (A) Lipofection of AlexaFluor488 labelled siRNA in a primary culture of rat dorsal root ganglion cells. Neuron in centre of the field is associated with siRNA on the membrane or within cytoplasm. Scale bar = 50 μm. (B) Regularly occurring action currents recorded through a cell-attached patch in voltage-clamp mode, without any applied polarisation, (patch depolarisation during action potentials associated with outward capacity current), in a neuron transfected with siRNAs specific for K_V_1.1. (C) Just sub and supra-threshold responses (light and heavy traces) to long duration current pulses in neurons exposed to control siRNA or 3 sequence specific siRNAs, upper and lower panels, respectively. Neurons held at − 90 mV in current-clamp. In the just supra-threshold response, the action potential is recruited later than in control. (D) Exposure of a single neuron to 10 nM DTX-K gives rise to similar changes in voltage-threshold as the sequence-specific siRNA. (E) K_V_1.1 sequence specific siRNA transfected neurons associated with more negative voltage-threshold than in control (*p = 0.016, *n* = 8, 8, *t*-test) (F) acutely applied 10 nM DTX-K shifts voltage-threshold more negative than control (*p = 0.014, *n* = 7, paired *t*-test).

Neurons with associated siRNA were viewed using eipifluorescence optics. The siRNA appeared as either points of luminescence or as a non-homogeneous haze, often apparently confined only to a part of the cytoplasm (Fig. 2A). Neurons with associated fluorescence were selected for recording. As is usual, just before going whole-cell, the cell-attached configuration was achieved in voltage-clamp mode. In two neurons treated with sequence selective siRNA against K_V_1.1, and without applying any polarising current, action potential firing was spontaneous and recorded before membrane rupture, because the action currents generated could be recorded across the patch (Fig. 2B). This was not seen in control transfections and has never been seen in any other similar recordings we have made in rat or mouse primary sensory neurons without K_V_1.1 sequence selective siRNA. This finding strongly suggests that the siRNA must change neuronal excitability by effective K^+^ channel down-regulation, and also confirms that the transfection protocol was effective. More usually, the action of active siRNA was found to shift the voltage-threshold for action potential firing, and to cause the recruitment of action potentials at longer latencies (Fig. 2C), consistent with the loss of a K^+^ current capable of dampening a depolarisation occurring at membrane potentials near − 60 mV. This is revealed by the difference in voltage-threshold between control and K_V_1.1 sequence selective siRNA treated neurons (Fig. 2E), where voltage-threshold was estimated as the most negative potential where the response to a depolarising current becomes non-passive and results in the production of an action potential (as described in e.g. Östman et al., 2008). Acute exposure to 10 nM DTX-K produced very similar changes both in action potential recruitment and in voltage-threshold (Fig. 2 D,F). Therefore under normal circumstances the function of K_V_1.1 is likely to be to maintain the firing threshold close to − 53 mV (Fig. 2 E,F), by quashing small depolarisations and thus inhibiting spontaneous firing. Previous work has revealed another vital contributor to spontaneous electrogenesis arising at membrane potentials more negative than − 60 mV, whose implicit presence is revealed with loss of K_V_1.1, and this is a regenerative inward current that is very likely to be the tetrodotoxin (TTX)-sensitive, persistent Na^+^ current previously described in similar large sensory neurons (Baker and Bostock, 1997).

### Sequence specific K_V_1.1 siRNA exposure does not widen action potentials

**Fig. 3:**
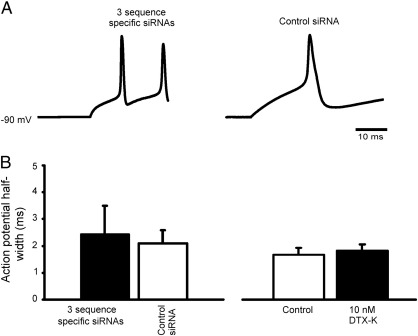
Action of sequence specific siRNA includes more negative voltage-thresholds although action potentials appear unaffected. (A) Left and right hand panels show example recordings from neurons transfected with 3 sequence specific siRNAs or control siRNA. Action potentials remain brief with sequence specific siRNA transfection, and in this example briefer than control. (B) Measurement of action potential half-width following transfection shows no significant effect of K_V_1.1 knock-down (*n* = 8, 8, n.s.), nor of acute exposure to 10 nM DTX-K in untransfected neurons (*n* = 6, n.s.).

Some neurons were highly repetitious, and fired trains of action potentials following transfection with sequence specific siRNA (similar to the effect of channel knock-out in auditory neurons (Brew et al., 2003)), but this was not always the case. The voltage-threshold for eliciting the first action potential evoked in an example sequence specific siRNA transfected neuron (Fig. 3A, left hand panel) was more negative than in control recordings (right hand panel), whereas the control action potential shown is clearly wider, suggesting a distinction between K^+^ channels controlling excitability and action potential width. Quantification of the action potential half-width in neurons exposed to sequence specific and control siRNA revealed that other K^+^ channel sub-types must be involved in the control of action potential duration, because action potentials could be wider in control recordings than following exposure to active siRNA and on average they were not different, Fig. 3B. Furthermore, acute exposure to 10 nM DTX-K did not alter action potential duration.

### Sequence specific siRNA knocks-down kcna1 mRNA assessed by qRT-PCR

**Fig. 4:**
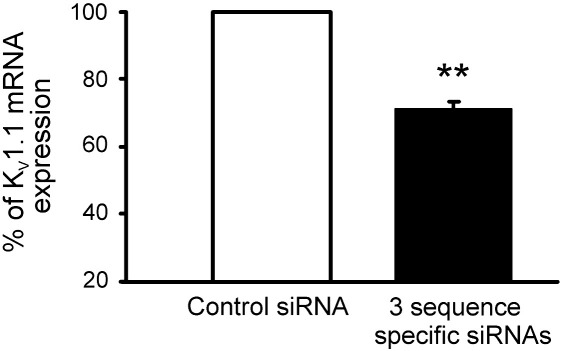
qRT-PCR confirms knock-down of K_V_1.1 following transfection with 3 sequence specific mRNAs. The amount of cDNA generated from the K_V_1.1 mRNA at 2 days following transfection was normalised to β-actin, mean fall in K_V_1.1 mRNA = 29. 67 ± 2.11%; **p = 0.005, one-sample *t*-test, indicative of a minimum 43% mRNA knock-down in neurons transfected with active sequence.

qPCR indicates a 30% reduction in the total K_V_1.1 message in sequence specific siRNA transfected cultures compared with control (Fig. 4; *n* = 3, *p* = 0.005). While this does not appear to be a substantial effect, in those neurons most adequately exposed to siRNA transfection, and subsequently selected for recording, the extent of knock-down must have been greater. This is because in the primary cultures many neurons are found clumped together and inaccessible to both the transfection reagents and patch-clamp, and secondly, not all accessible neurons were associated with fluorescence. Analysis of photomicrographs of primary cultures, taken following transfection for subsequent qRT-PCR, revealed 35 of 50 surviving neurons (> 25 μm in apparent diameter) appeared to be associated with fluorescence. Therefore 43% loss of the message represents the absolute minimum knock-down in transfected neurons. While it is impossible to know the proportion of the total number of neurons to be found in clumps, because they cannot be counted, it is probable that siRNA knock-down in transfected neurons is substantially greater than this.

### Functional effects of sequence specific siRNA in vivo

**Fig. 5:**
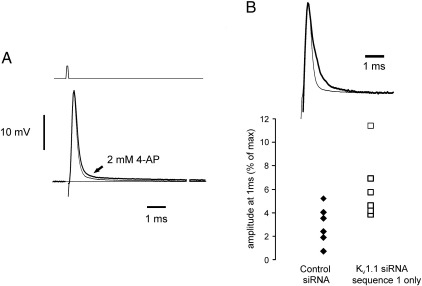
Blockade of kinetically fast delayed rectifier in myelinated spinal root axons increases the amplitude and duration of the DAP. (A) Application of a drop of 2 mM 4-AP in extracellular solution to a spinal root causes an increase in the DAP following a maximal amplitude compound action potential (control and with 4-AP, light and heavy lines, respectively) from a relative amplitude of 3.7 to 5.6% at 1 ms following action potential peak, in this example. (B) Pre-exposure to intrathecal siRNA causes a similar small increase in the relative amplitude of the DAP at 1 ms (raw data shown, control = 2.97 ± 0.66, K_V_1.1 siRNA = 6.08 ± 1.15% mean ± s.e.m. *p* < 0.02, Mann–Whitney *U*-test).

*In vivo* transfection was carried out with only one sequence specific siRNA, sequence 1, in part to reduce cost, although sequence 1 was also shown to be effective *in vitro* in shifting the voltage-threshold in cultured neurons. A modest increase in the average normalised amplitude of the DAP occurring immediately after a maximal compound action potential was observed in the K_V_1.1 siRNA treated group in comparison with control, Fig. 5, a finding consistent with a loss of some K^+^ channel function in A-fibre internodes. The effect of 4-AP on the DAP was also recorded in similar preparations, where 4-AP in the uncharged form is expected to be able to penetrate myelin sheaths to access the internodally expressed K^+^ channels underneath. As previously reported, intermodal K^+^ channel block by 4-AP enhanced the DAP.

## Discussion

K_V_1.1 channel knock-down by siRNA's targeting the *kcna1* mRNA *in vitro* has been shown in the following two ways. Firstly, our functional data clearly show that the excitability of neurons is increased, with a statistically significant movement of voltage-threshold to more negative potentials. Consistent with this finding is the induction of resting spontaneous action potentials without the pre-requisite of intracellular dialysis on going whole-cell, and not seen in other circumstances. This suggests that the voltage-threshold is either at, or more negative than, the membrane potential. Secondly, qRT-PCR indicates a significant reduction of *kcna1* mRNA in cultures treated by siRNA targeting the channel message. Exposure to siRNA *in vivo* caused an increase in the amplitude of the early DAP following a maximal compound action potential, explained by either an increase in internodal membrane resistance and/or a quite subtle widening of the compound action potential. Such a widening has been reported before (Bostock et al., 1981) when spinal root axons were exposed to 4-AP, and may be related to conduction velocity changes within the envelope of the compound potential. However, either phenomenon may be related to functional changes in K^+^ channels.

The action of DTX-K on sensory neuron excitability reported here is consistent with the block of a K^+^ current primarily responsible for controlling membrane conductance at potentials near rest. The DTX-K sensitive current is a fast delayed rectifier, kinetically similar to the current produced by heretologous expression of K_V_1.1 (Grissmer et al., 1994). The knock-out of K_V_1.1 produces well described effects on excitability in peripheral motor nerve (Zhou et al., 1998), precipitates seizures by reducing K^+^ currents in the hippocampus (Smart et al., 1998; Wenzel et al., 2007), and enhances inflammatory hyperalgesia (Clark and Tempel, 1998). But while the effects of a selective K_V_1.1 down-regulation in primary sensory neurons have not been well described, the expression of dendrotoxin sensitive K^+^ channels in similar neurons has been known for many years (e.g. Stansfeld and Feltz, 1988). Although we have not made an exhaustive study of the effects of K_V_1.1 block or knock-down on action potential duration, it is clear that in the sub-population of neurons we have studied, other K^+^ channels must make a major contribution to determining the shape of the action potential. This is because action potential duration is not significantly longer after K_V_1.1 siRNA exposure than in controls, and acute exposure to DTX-K does not have a major effect on action potential duration (while at the same time voltage-threshold and repetitiousness are altered by both our siRNA and DTX-K). Therefore, in the cell body, K_V_1.1 must contribute to channels primarily controlling the threshold and accommodative properties of the sub-population of sensory neurons we studied, a conclusion very similar to that reached for the effect of DTX-α on nodose A-type neurons (where the α-toxin does not discriminate well between K_V_1.1, 1.2 and 1.6, Glazebrook et al., 2002), and analogous to the role of I_KL_ in auditory neurons (Brew et al., 2003). Given the circumscribed expression pattern of *Shaker* homologue K^+^ channels in axons (Wang et al., 1993; Rasband et al., 1999), under the myelin, and our present *in vivo* data with siRNA administration, K_V_1.1 is indeed likely to control the amplitude of the depolarising after potential (DAP) in axons, and hence contribute to the accommodative properties of sensory A-fibres (Baker et al., 1987).

Knock-down of K_V_1.1 expression produces an effect on neuronal excitability qualitatively similar to exposure to DTX-K. This is an interesting finding because in order to be subject to toxin block, the channel is thought to necessarily incorporate only one K_V_1.1 sub-unit (Wang et al., 1999). Making the assumption that the sensory neurons we have studied are unlikely to express only one K_V_1 channel sub-family member, the change in voltage-threshold we have observed with siRNA implies that K_V_1.1 expression may therefore be necessary for normal channel function or expression, and its presence within a heteromultimer may not be redundant (Wenzel et al., 2007).

Large primary sensory neurons generate persistent Na^+^ currents over a wide range of membrane potentials, including, for example, at − 70 mV (Baker and Bostock, 1997). These currents are blocked by sub-micromolar concentrations of tetrodotoxin (TTX), and are therefore not generated by the TTX-resistant channel Na_V_1.9, which is a marker for nociceptors (Fang et al., 2006) and predominantly expressed with Na_V_1.8 (Abrahamsen et al., 2008). Our previous explanation for their generation is that they probably arise from unusual gating patterns of transient Na^+^ channels expressed in the same neurons (that includes a shift in activation voltage-dependence to more negative potentials and loss of normal inactivation). Such ‘modal-gating’ has been well documented (e.g. Böhle and Benndorf, 1995; Alzheimer et al., 1993; Baker and Bostock, 1998). Similar persistent Na^+^ currents are almost certainly responsible for the pacemaker current driving ectopic action potential generation in demyelinated peripheral nerve (Baker and Bostock, 1992), that is revealed by exposure to 4-AP. Our expectation is that the loss or reduction of K_V_1.1 in our present experiments allows this persistent Na^+^ current to drive spontaneous discharge in sensory neurons (c.f. Fig. 2), consistent with the normal function of the K_V_1.1 current being to suppress such activity, and therefore to be critically involved in the control of threshold.

The original reasoning for the use of 4-AP (fampridine) in the treatment of demyelinating disease was the expectation that it should widen action potentials and so increase impulse conduction safety factor, where there was internodal membrane involvement in impulse transmission (Sherratt et al., 1980; Bostock et al., 1981). In fact, the clinically useful concentrations of 4-AP are likely to be too low for this to occur (Smith et al., 2000). However, evidence does exist for changes in membrane excitability in neurons or axons (Smith et al., 2000) when 4-AP is administered systemically. Because 4-AP produces a voltage-dependent block of K^+^ channels (e.g. Howe and Ritchie, 1991), those operating around the resting potential will be particularly susceptible to block, and K_V_1.1 must be included within this group. However, block is reduced at more depolarised potentials, for example, during an action potential. Under special circumstances where the major voltage-gated K^+^ conductance is provided by channels incorporating K_V_1.1, (and this may well be the case in demyelinated axons), this channel would be expected to contribute to the control of action potential duration. Our findings might point to an alternative route to selective knock-down for a K^+^ current component in dorsal column axons (for example) to test the possibility that having wide axonal action potentials following demyelination might be functionally and behaviourally beneficial.

## Experimental methods

### Tissue culture and transfection *in vitro*

Dorsal root ganglion (DRG) neurons were isolated from Wistar rats (> P21, ~ 75–150 g) killed in accordance with Home Office guidelines by cervical dislocation. In the experiments involving siRNA, the rats were exclusively male. Neuronal cultures were prepared as described previously (Baker et al., 2003; Baker and Bostock, 1997), replacing Dispase in the dissociation media with Sigma type IX protease (1 mg ml^− 1^), and with the modification of passing the dissociated DRG tissue suspension through a 100 μm cell filter (BDH Falcon, VWR, Lutterworth, UK) before centrifugation and plating-out on 13 mm glass coverslips (coated using poly-l-lysine, (Sigma-Aldrich, Poole, UK)) inside 35 mm diameter Petri dishes (Primera, Easy grip, (Falcon)) in normal media (DMEM low glucose (PAA, Yeovil, UK)) + 5% Pen Strep (10,000 U penicillin + 10 mg streptomycin ml^− 1^ (Sigma Aldrich) + 10% heat inactivated FBS (PAA)).

The original siRNA samples were prepared in annealing buffer according to the manufacturer's instructions (Qiagen, Crawley, UK). After maintaining the neurons overnight in an incubator at 37 °C with a 5% CO_2_ atmosphere, siRNA was transfected using oligofectamine (Invitrogen, Paisley, UK). In experimental cultures, 3 different siRNA duplexes targeting K_V_1.1 were transfected together, one of which was fluorescently tagged on the sense-strand (3′-AlexaFluor 488), to enable the subsequent visualisation of neurons associated with the siRNA (Fig. 2). Other reports exist in the literature where more than one siRNA are used for transfection without testing each individual efficiency (e.g. Bertram and Hass, 2008), and previous experiments have employed simultaneous transfection of multiple siRNAs to knock-down expression of the K^+^ channel, Eagl (Weber et al., 2006). Parallel control transfections were also performed using siRNA targeting no known sequence (All Stars, Qiagen; e.g. Bertram and Hass, 2008). The control siRNA was also fluorescently tagged (3′-AlexaFluor 488). All transfections employed the same amount of siRNA (53 pmol per dish in a total final volume of 0.75 ml OptiMEM media, Invitrogen), giving a final concentration very close to 71 nM, and 5 μl of oligofectamine. After 4–5 h incubation at 37 °C, 325 μl of OptiMEM with 30% FBS (PAA) was added, and the following day replaced with normal media.

The anti-sense strand sequences of the target specific siRNA were:

1. r (UUAAACAUCGGUCAGGAGC)dTdT
2. r (UGUGGUGUUAUCGAUGCGG)dTdG
3. r (AUACAAUGGCAAUAACCCG)dTdG.

After investigating the effects of 3 simultaneously transfected siRNAs, the effect of a single siRNA (sequence 1) was also studied.

### Electrophysiology and pharmacology in vitro

The effects of the siRNA on the electrophysiological properties of a sub-population of surviving neurons were investigated 2 days following transfection. The sub-population of interest was characterised as being composed of neurons > 25 μm in apparent diameter and generating uninflected action potentials, allowing the probable exclusion of Na_V_1.8 expressing nociceptors. The whole-cell current-clamp methodology employed, and voltage-threshold estimation technique, have been described in detail elsewhere (e.g. Baker, 2005; Östman et al., 2008). External and internal current-clamp solutions contained the following (in mM). Extracellular: 140 NaCl, 10 HEPES (hemi-Na), 2.1 CaCl_2_, 2.12 MgCl_2_, and 2.5 KCl. Intracellular: 143 KCl, 3 EGTA-Na, 10 HEPES (hemi-Na), 1.21 CaCl_2_, 1.21 MgCl_2_, 3 ATP (Mg), and 0.5 GTP (Li). Solutions were buffered to pH 7.2–7.3 with small additional amounts of NaOH or HCl. In order to measure the effect of channel block on excitability, current-clamp experiments were carried out where 10 or 100 nM dendrotoxin (DTX-K) (Alomone Labs) was applied to neurons by local superfusion. The toxin was made-up in 0.01% BSA containing external solution. In a separate series of experiments, untransfected neurons were exposed to 10 or 100 nM DTX-K and the effect of toxin on the whole-cell outward currents recorded in voltage-clamp. The external and internal solutions used for voltage-clamp were the same as those used for whole-cell current-clamp, with the addition of 250 nM tetrodotoxin (TTX, Alomone Labs) to the external solution, that blocked Na^+^ currents. All recordings were made at room temperature, 20–22 °C.

### Action potential half-width measurements

Action potential half-width measurements were made in current-clamp, where a just suprathreshold stimulus was applied. The action potential duration was estimated by placing vertical cursors in Clampfit (Molecular Devices) at the points where the rising and falling phases intersect the membrane potential mid-way between the holding potential and the peak.

### qRT-PCR

Parallel cultures were treated identically and transfected at the same time with either the 3 K_V_1.1 targeting sequences or with All Stars negative control siRNA, as above. mRNA was extracted from 3 experimental and control primary cultures, 2 days following transfection, where the DRG tissue used derived from a total of 5 adult Wistar rats. The proportion of surviving neurons associated with fluorescence at 2 days post transfection was estimated by photographing cell fields where neurons were abundant under high power for both control and K_V_1.1 siRNA transfected cultures, and counting those neurons greater than 25 μm in apparent diameter found, either with, or without, associated fluorescence.

The DRG cultures were scraped-off the culture dish and resuspended in RNA isolation buffer (RNeasy; Qiagen). RNA (1 μg) was reverse transcribed with a cDNA synthesis kit (Invitrogen) and expression levels measured by qRT-PCR on a StepOnePlus machine (Applied Biosystems), utilising Fast SYBR Green Master Mix (Applied Biosystems). The housekeeping gene β-actin was also amplified, and used to normalise the amount of cDNA product for K_V_1.1 (c.f. Shao et al, 2009). Briefly, the reaction was performed in a total reaction volume of 20 μl, consisting of 10 μl Fast SYBR Green Master Mix and including a final concentration of 0.5 μM of primer mix for K_V_1.1 (0.2 μM for β-actin), and 400 ng cDNA. All reactions were performed in triplicates for each sample. For data processing and identifying the percentage of expression for each gene-interested, the threshold amplification cycles (C_T_) were determined using StepOnePlus software, and analysed by the 2^−∆∆C_T_^ method (Livak and Schmittgen, 2001).

The primers used for amplifying K_V_1.1 mRNA were as follows:

f 5′-CCACTACAGGCAGGCTAATATCAG-3′
r 5′-TGATCAGTTGCGGTGCAGTT-3′.

The primers used for amplifying β-actin were as follows:

f 5′-TCCTGTGGCATCCATGAAACT-3′
r 5′-AACGTCACACTTCATGATGGAATT-3′.

### Transfection in vivo

The *in vivo* transfection procedure was performed in male adult Wistar rats (330–430 g) anaesthetised using isoflurane, by acutely cannulating the intrathecal space at the L5–L6 vertebrae, and pushing sterile 0.7 mm outer diameter PET tubing (Merck Clevenot) 1.5 to 2 cm caudally within the sacrum. The bone between the spinous processes was exposed using the minimum displacement of muscle, and a hole created at the midline L5–L6 border using a hypodermic syringe. The benefits of cannulating low down in the spine were two-fold. Firstly, when the cannula entered the spine it did so below the level of the spinal cord, into the fluid filled cavity. Secondly, the siRNA was released in the vicinity of the sacrum, close to the cell bodies supporting the longest dorsal roots within the cauda equina, and the most suitable for subsequent recording. Over a period of about 1 min, 30 μl of sterile buffer containing 9.3 μg of either sequence 1 or control siRNA, and 12 μl of oligofectamine was injected through the cannula using a Hamilton syringe. The injected solution had the following composition: 3 μl siRNA (20 μM), 12 μl oligofectamine, 15 μl sterile buffered saline solution (Fluka Ultra 2×). Some fluorescent solution leaked from the spine when the cannula was withdrawn. The animals were allowed to recover in a warmed cage, and subsequently survived for 2 days before spinal root recording under terminal anaesthesia.

### Recording from spinal roots

Rats previously infused intrathecally were anaesthetised using Domitor (medetomidine)/Ketamine (0.5 mg/kg and 75 mg/kg, respectively, IP). Following a laminectomy, monophasic action potentials were recorded from sacral roots that were supported by two gold-plated hook electrodes, positioned at the proximal and distal ends, while the root was bathed in a moist paraffin pool created above the spine (cf Baker and Bostock 1989). The distal end of the root remained in continuity, and could be stimulated supramaximally by a constant current pulse, while the central end was cut close to the root entry into the cord and crushed just distal to the proximal gold plated electrode. A fine tipped, non-polarisable glass electrode containing KCl agar was then brought in contact with the root mid-way between the gold-plated electrodes, and the extracellular membrane potential recorded between the glass electrode and the proximal cut end using a high-impedance differential amplifier (DP-311, Warner Instruments, Harvard Bioscience, Kent UK).The temperature of the paraffin pool was maintained close to 30 °C using an incandescent lamp, and measured using a thermister in the paraffin. The root was stimulated using a 100 μs constant current pulse (Neurolog NL800A, Digitimer, Welwyn Garden City, UK) between the distal electrode and ground. Triggered recordings of action potentials were recorded on-line using a 1401 plus and signal software (CED, Cambridge UK), running on a Dell PC, filtered at DC — 10 kHz or 0.1 Hz–10 kHz and sampled at 20 kHz.

### Data analysis

Wherever possible, data are presented as mean ± s.e.m. Statistical comparisons are made using the unequal variance *t*-test, paired *t*-test, one-sample *t*-test, Mann–Whitney *U*-test and one-way, repeated measures ANOVA, as appropriate.

## Conflict of interest

The authors report no conflict of interest and are students or employees of Queen Mary University of London or Imperial College London.
